# Causality Assessment Guidelines for Adverse Events Following Immunization with a Focus on Guillain–Barré Syndrome

**DOI:** 10.3390/vaccines8010101

**Published:** 2020-02-24

**Authors:** Hankil Lee, Hye-Young Kang, Sunghwa Cho, Seonyoung Park, Ah-Young Kim, Sun-Young Jung, Baik Lin Seong, Young-Mock Lee

**Affiliations:** 1 Division of Pharmaceutical Outcomes and Policy, UNC Eshelman School of Pharmacy, University of North Carolina at Chapel Hill, Chapel Hill, NC 27599, USA; hankil912@gmail.com; 2 College of Pharmacy, Yonsei Institute of Pharmaceutical Sciences, Yonsei University, Incheon 21983, Korea; hykang2@yonsei.ac.kr; 3 Department of Pharmaceutical Medicine and Regulatory Sciences, Colleges of Medicine and Pharmacy, Yonsei University, Incheon 21983, Korea; sarah140@yonsei.ac.kr (S.C.); psy892002@hotmail.com (S.P.); pharmay@yonsei.ac.kr (A.-Y.K.); 4 College of Pharmacy, Chung-Ang University, Seoul 06974, Korea; jsyoung01@gmail.com; 5 Department of Biotechnology, College of Life Science and Biotechnology, Yonsei University, Seoul 03722, Korea; blseong@yonsei.ac.kr; 6 Department of Pediatrics, Yonsei University College of Medicine, Seoul 03722, Korea

**Keywords:** adverse event, causality assessment, guideline, Guillain–Barré syndrome, vaccine

## Abstract

South Korea operates a National Vaccine Injury Compensation Program (VICP) for people who experience adverse events following immunization (AEFI). To run this program rationally, it is a prerequisite to confirm whether adverse events were caused by immunization. Guillain–Barré syndrome (GBS), a severe neurological disease with limb pain and muscle weakness as cardinal symptoms, is attracting attention as an AEFI. However, algorithm or guidelines for assessing the causality between vaccination and the incidence of GBS are lacking. We aimed to develop guidelines for causality assessment of GBS as an AEFI and suggest using these guidelines in alignment with the VICP. We systematically searched for other previously published algorithms or guidelines and found a WHO-AEFI guideline used worldwide; however, it only provides general instructions and is not tailored to specific adverse events. We translated and locally adapted the structure of this guideline and then added contents related to GBS. The GBS-specific guideline consists of four steps: case ascertainment of GBS, checklist (including (1) order of incidence, (2) temporal proximity, (3) evidence for other causes and (4) published evidence), an algorithm, and final classification. We listed key information on confirming GBS and whether any other causes of GBS were present. For real world application of the guideline along with the VICP, we collaborated with a panel of neurologists, epidemiologic investigators, and committee members from the VICP. To ensure transparency and a scientific approach, regular updates and collaboration with neurologists are essential. We expect that this guideline will contribute to logical causality assessment and compensation decisions for GBS and will provide the basic structure for causality assessment of other AEFIs.

## 1. Introduction

South Korea’s national Vaccine Injury Compensation Program (VICP) is implemented by the Korea Centers for Diseases Prevention and Control (KCDC) for damage associated with adverse events following immunization (AEFI) and is covered by the National Immunization Program (NIP) [1]. Adverse events (AEs) following immunization are defined as any untoward medical occurrences that follow immunization and do not necessarily have a causal relationship with the vaccine usage [2]. To compensate medical and/or financial damage following vaccination, it is necessary to that the AEFI were caused due to vaccination. If the causality assessment is not scientific or objective, the assessment results will be inconsistent, and consequently the compensation program will no longer have credibility. Thus, it is crucial to have a valid causality assessment system for better operation of the compensation program.

There are a number of causality assessment tools for AEs caused by drugs, including the World Health Organization–Uppsala Monitoring Center (WHO-UMC) system [3,4], Naranjo algorithm [5], Modified Kramer Method [6], Roussel Uclaf Causality Assessment Model (RUCAM) algorithm [7], and Alden algorithm [8]. By comparison, a systematic review has identified only one tool for causality assessment of vaccines, developed by the World Health Organization (WHO). This user manual was developed in 2013, and the second edition was published in 2018 [9]. According to the WHO, it was intended to be used by staff at national and subnational levels (such as members of the national AEFI committee or immunization program managers) as a guide to a systematic and standardized global causality assessment process for individual serious AEFI [9]. The WHO user manual provides useful guidelines for causality assessment of AEFI and presents the following standardized steps: eligibility, checklist, algorithm, and classification. However, they are general guidelines for all types of AEFI and not specific to individual types of AEFI. The types and strength of evidence and factors needed for causality assessment may be different depending on the type of AEFI. AEFI-specific causality assessment guidelines would be helpful for conducting more accurate causality assessments.

Therefore, our study aimed to develop AEFI-specific causality assessment guidelines using the basic framework suggested by the WHO manual. In particular, we developed causality assessment guidelines for Guillain–Barré syndrome (GBS), a severe neurological AEFI, with limb pain as the major symptom [10]. GBS is not a common AEFI but has received special attention due to the seriousness of its symptoms and sequelae. The background incidence of GBS is estimated at 0.8–1.9 per 100,000 person-years and increases with age [11]. The incidence of GBS following vaccination varies according to the flu season, and it is known that an additional 1 to 2 cases of GBS occur after 1 million doses of the influenza vaccine [12]. Although we developed these guidelines in Korea, we expect they will be generalizable to other countries because they were developed on the basis of the WHO framework and international literature and evidence.

## 2. Materials and Methods

**Figure 1:**
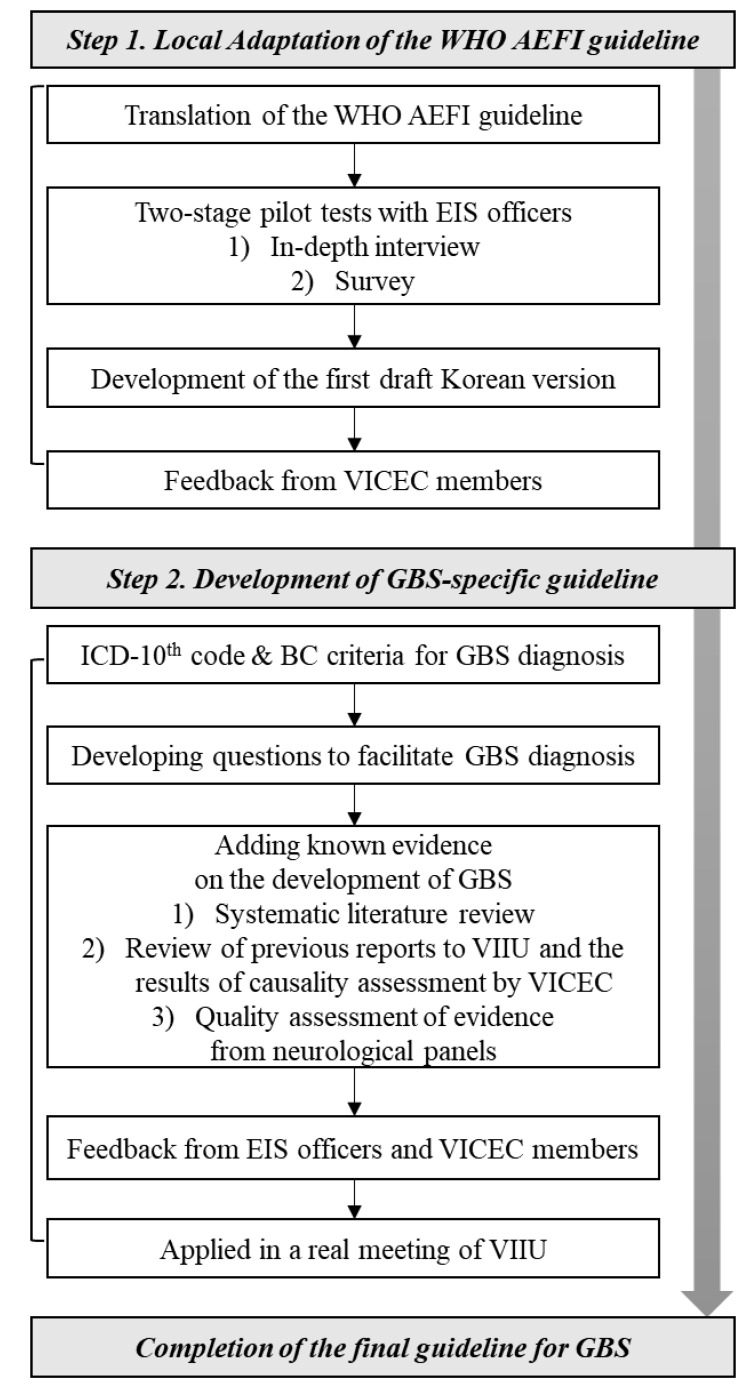
Steps involved in developing a causality assessment guideline for Guillain–Barré syndrome following immunization. (AEFI: Adverse Events Following Immunization; BC: Brighton Collaboration; EIS: Epidemiology Intelligence Service; GBS: Guillain–Barré syndrome; VICEC: Vaccine Injury Compensation Expert Committee; VIIU: Vaccine Injury Investigation Unit).

We developed the causality assessment guidelines for GBS following immunization in two steps: local adaptation of the global WHO guidelines and development of GBS-specific guidelines (Figure 1).

### 2.1. Local Adaptation of the Global WHO Guidelines

First, we translated the “Annex 1: Worksheet for AEFI causality assessment” from the second edition of the WHO user manual for AEFI causality assessment [9] into Korean and developed a draft Korean version. We followed the same structure of steps for causality assessment of an individual AEFI included in the WHO manual. They are as follows: eligibility, checklist, algorithm, and classification. All items presented with these steps were incorporated.

Eligibility (step I) aims to determine if the AEFI case satisfies the minimum criteria for causality assessment. Questions to ascertain the eligibility include “Was the vaccine administered before the event occurred?”, “What is a valid diagnosis?”, and “Did the diagnosis meet a case definition?” In step I, the investigator is also asked to create his or her question on causality such as “Has the vaccine A caused GBS?” Checklist (step II) involves systematically reviewing the relevant and available information to address possible causal aspects of the AEFI. Questions included in the checklist are as follows: “Is there strong evidence for other causes?”, “Is there a known causal association with the vaccine or vaccination?”, ‘Was the event within the time window of increased association risk?’; “Is there strong evidence against a causal association?”, and “Are there other qualifying factors for classification?” Some of the question categories also consist of detailed sub-questions. An Algorithm (step III) identifies whether there is a trend suggesting causality using the information gathered in the checklist. On the basis of the trend determined in the algorithm, classification (step IV) categorizes the AEFI association with the vaccine, or vaccination, into four categories. A case with adequate information for a causality conclusion can be classified as one of the following three categories: consistent causal association with immunization, indeterminate, and inconsistent causal association with immunization (coincidental). A case without adequate information could additionally be classified as “unclassifiable”.

Second, for the local adaptation of the WHO manual, we conducted two-stage pilot tests with Epidemiology Intelligence Service (EIS) officers. Because EIS officers are in charge of collecting detailed information about the AEFI as the initial step of the VICP (e.g., patient history and physical findings) and are responsible for filling out the checklist from the guidelines, we considered that their feedback was crucial in developing a locally adoptable guideline. As the first stage, we conducted an in-depth interview with two EIS officers (one had many years of experience as an EIS officer and the other was a pediatric specialist) to collect unstructured feedback on the draft of the translated guideline. This focused on the structure of the guideline, terminology, appropriateness, and feasibility of the individual items included in the draft. Based on their feedback, we revised the draft. In the second stage, we conducted a pilot test of the revised Korean version with seven EIS officers, who attended a Vaccine Injury Investigation Unit (VIIU) meeting during the study period. We asked the participants to apply the draft Korean version to AEFI cases that the Vaccine Injury Compensation Expert Committee (VICEC) had previously reviewed. Based on their feedback about the readability, appropriateness, and feasibility of individual items and their overall opinions, a further revision was made, and the general causality assessment guidelines were finalized.

### 2.2. Development of GBS-Specific Guidelines

From the Korean version of the general guidelines, we developed a set of items tailored to GBS cases. GBS is a neurological disorder accompanied by paralysis, and a precise diagnosis is a paramount first step in causality assessment because many diseases have similar symptoms. We first defined GBS using the Brighton Collaboration (BC) criteria [2]. Subsequently, further questions aimed at ascertaining a GBS diagnosis were developed by reviewing previous studies [13,14,15] and the medical records attached to GBS cases previously identified as AEFI, which had been reported to the VIIU.

We then focused on the assessment of temporal proximity and other known causes of GBS. In order to develop evidence-based items, we conducted a systematic literature review (Supplementary S1). We also examined all previous reports to the VIIU as well as the results of the VICEC’s causality assessment for GBS and extracted suspected individual causes of GBS. Then, we qualitatively synthesized evidence and references as a result of the quality assessment by 10 neurologists (Supplementary S2). Finally, by customizing all remaining items in the checklist, the first draft of the GBS-specific causality assessment guideline was complete. Next, we proceeded to ask three neurologists about the clinical validity of the first draft again. Based on their feedback, we revised the guidelines and prepared the second draft. We then asked seven epidemiologists about its feasibility of the second draft. The third draft of the GBS-specific guidelines was then applied in an actual meeting of the VIIU as a pilot test, and the final version was completed by reflecting on feedback from the committee members of the pilot test.

## 3. Results

### 3.1. Structure of the Guidelines

**Figure 2:**
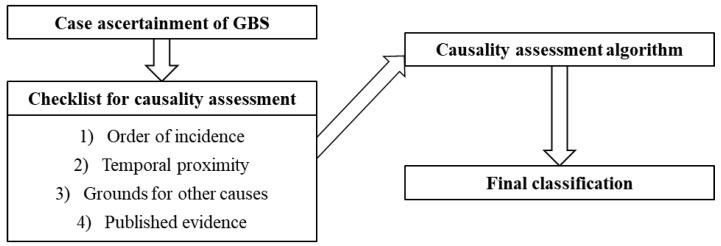
Structure of the causality assessment guidelines for Guillain–Barré syndrome following immunization.

The guidelines developed in the present study are based on the structure of the WHO AEFI guidelines and includes the following: case ascertainment of GBS; checklist for causality assessment composed of (1) order of incidence, (2) temporal proximity, (3) grounds for other causes, and (4) published evidence; causality assessment algorithm; and final classification (Figure 2).

### 3.2. Case Ascertainment of GBS (Eligibility)

Case ascertainment of GBS is not a part of causality assessment, but it is an important prerequisite task that triggers causality assessment. Diagnosis of GBS is confirmed by G61.0 of ICD-10th code and BC criteria (Table 1). These are sets of standard diagnostic criteria for GBS that use 4 levels of classification, with “level 1” indicating the highest level of diagnostic certainty (definite). For each patient with GBS, the reviewer fills in the corresponding checkboxes for each criterion and decides the level of diagnostic certainty. Since it is not routine clinical practice to collect all the information included in BC criteria, depending solely on these criteria to ascertain GBS is unrealistic. Therefore, our study developed additional questions to help with diagnosis: “Was the paralysis ascending?” and “Did it take less than 4 weeks for symptoms to stop worsening?” (Table 1). The more questions that are answered with “Yes”, the more likely the diagnosis of GBS is correct. We also added four questions that might indicate diseases other than GBS. For these questions, the more questions that are answered with “Yes”, the less likely the diagnosis of GBS is correct.

### 3.3. Checklist

To address the possible causal relationship between vaccination and GBS, a number of questions were included in the checklist (Table 2). Questions were grouped into the following four themes: order of incidence, temporal proximity, evidence for other causes, and published evidence of a causal association. Order of incidence asks whether the vaccine was administered before GBS onset. Temporal proximity intends to confirm whether GBS occurred within a plausible time window after vaccine administration. According to a literature review, the incidence of GBS occurring within 6 weeks following immunization is considered to have causality with the vaccination [16,17].

To examine the evidence against a causal association, questions regarding various causes other than the vaccine were included in the checklist. As a first step, the patient’s GBS and vaccination history were reviewed. Reviewing patient’s history helps decide whether this occurrence of GBS is due to vaccination, or whether other causes should be investigated.

Based on a literature review and consultation with the clinician panel, we found that several conditions are known to cause GBS [18]. These include upper respiratory infection [18,19,20,21], gastrointestinal disorders [18,19,20,22], Zika/Dengue infection [23], Malaria infection [24,25], Tsutsugamushi infection [26], surgery history [27,28], campylobacter infection [29,30,31,32,33,34], cytomegalovirus infection [33,34,35,36,37,38], Epstein–Barr virus infection [33,38,39,40,41], herpes simplex virus [39], varicella-zoster virus infection [42], mycoplasma pneumonia infection [43,44], haemophilus influenza infection [18,45], and influenza virus infection [46,47]. For each condition, the checklist asks whether the patient had the condition prior to the manifestation of GBS. Most members of the clinical panel agreed that the chosen time window of 6 weeks was appropriate for assigning a causal association of GBS with prior infections. Our guideline provides instructions to write the date of prior infections in the note column of the checklist.

In the last part of the checklist, the guideline provides up-to-date published evidence about the causal association between various vaccines and GBS. The vaccines included are influenza, meningococcal, MMR, polio, HPV, hepatitis A, hepatitis B, and varicella vaccine. The evidence could either support or oppose the causal association. For each vaccine, the most up-to-date evidence should be provided, so that the causality assessment is performed on the basis of the most current evidence.

### 3.4. Algorithm and Classification

**Figure 3:**
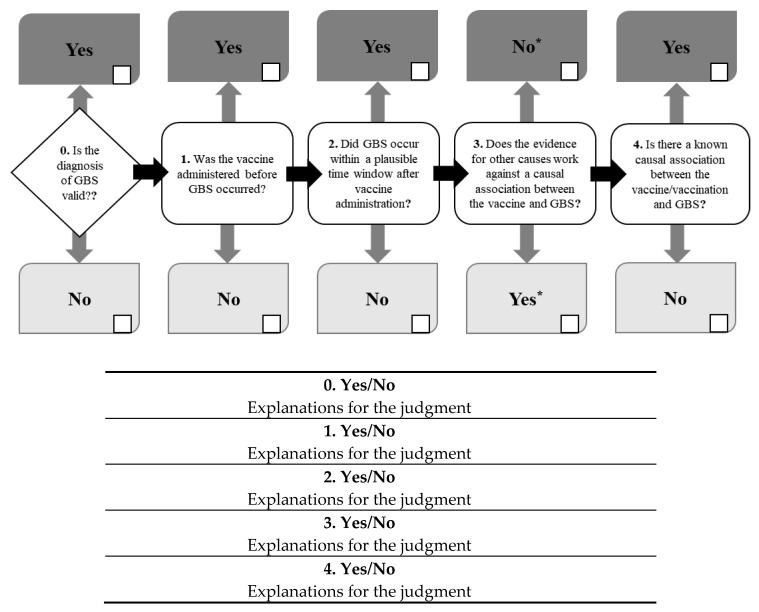
Causality assessment guidelines for Guillain–Barré syndrome following immunization: Causality assessment algorithm. (* Unlike other questions, for this question, “No” implies a causal association, and “Yes” implies little causal association.).

Based on the information collected in the checklist, the reviewer answers each of the five questions included in the causality assessment algorithm as “Yes” or “No” (Figure 3). The first question was numbered as “0” because a valid diagnosis of GBS is a pre-requisite condition to proceed with the causality assessment algorithm. As the number of questions with positive answers (“Yes”) for the causal association increases, causality between the vaccination and GBS increases. As an exception, a negative answer (“No”) to the question “Does the evidence for other causes work against a causal association between the vaccination and GBS?” is considered as a positive answer. For all questions, we are required to provide explanation for a positive or negative answer.

**Figure 4:**
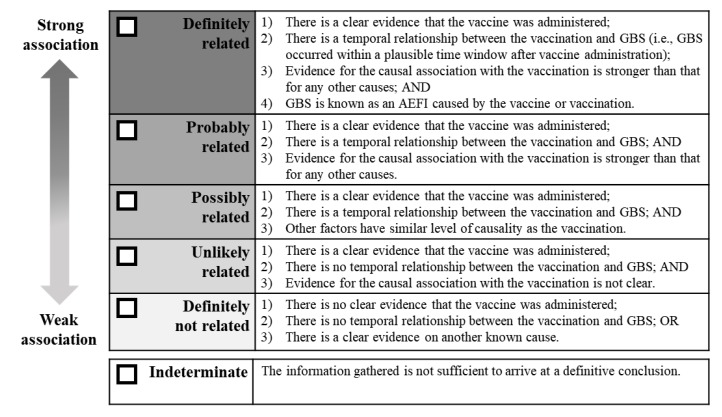
Causality assessment guideline for Guillain–Barré syndrome following immunization: classification of the extent of the causal association.

The last step is to classify the extent of GBS’s association with the vaccine, or vaccination, on the basis of the trend determined by the algorithm and to arrive at a decision on causality. The extent of the causal association is classified into the following five categories: “definitely related,” “probably related,” “possibly related,” “unlikely related,” and “definitely not related.” The framework of this classification follows that of the pre-existing framework by the Korean VICP. A separate classification expressed as “indeterminate” is also included. After completing the checklist and using the algorithm, the reviewer may discover that the information gathered is not sufficient to arrive at a definite conclusion. As such, the case can be classified as “indeterminate,” and requires the reviewer to specify the missing information that prevents the classification of the case. Detailed criteria for each classification are presented in Figure 4.

### 3.5. Application

We recommend that the guidelines developed in this study should be used to assess causality in the VICP in Korea as follows. First, when GBS is reported as an AEFI, the EIS officer who is in charge of collecting basic information about the AEFI, fills out the checklist as the first official step of the VIIU. Second, the Investigation Committee of the VIIU reviews the checklist and revises it if necessary. Based on the revised checklist, the officers use the causality assessment algorithm and determine a draft classification of the AEFI, according to one of the six categories. Lastly, the VICEC reviews the answers of each question in the algorithm and the classification drafted by the Investigation Committee on VIIU and arrives at a decision on causality. Based on the causality decision, the eligibility of AEFI for compensation is determined.

In addition, the context is important and necessary for the guidelines to be effective. First, the guidelines developed in this study were based on the most recent literature and scientific evidence available at the time of the study. To maintain the effectiveness of the guidelines, we recommend that the VIIU continues to collect new evidence, establishes a database of the evidence, and regularly updates the guidelines. Second, EIS officers play a significant role in ensuring the guidelines perform as intended. This is because they are at the forefront in collecting information about AEFI from the clinical field; therefore, they can provide the key information for causality assessment. Furthermore, they are the first people who fill out the checklist, which is the starting point of the causality assessment process. Thus, EIS officers should undergo proper training on how to use the guidelines. Establishing a database for the literature on the association of GBS with vaccination and continuous updating of this database will be crucial in helping EIS officers to perform their duties efficiently. Third, unlike other types of AEFI, neurologic AEs, such as GBS, are challenging in terms of achieving valid diagnoses due to the nature of symptoms and clinical features. In the process of guideline development, consultation with neurologists was crucial in developing checklist items used to ascertain GBS. Therefore, we recommend that the VIIU utilize a consultation panel composed of neurologists who will be actively involved throughout the causality assessment process.

## 4. Discussion

### 4.1. First Guidelines for Causality Assessment between Guillain–Barré Syndrome and the Vaccine Following Vaccination

This study was conducted to contribute to objective and valid causality assessment by developing guidelines to assist in determination of causality between GBS and vaccination. The WHO AEFI guidelines were developed as a tool to assess the causality of AEFI; however, they are insufficient for assessing individual AE because they only present the overall framework and flow. Pathological mechanisms of action, as well as the relationship between the patient’s underlying disease and an AE, should be closely examined to determine causality. Accordingly, effective guidelines for the causality assessment of AEFI must include items related to the specific AE; thus, there is a need for causality assessment guidelines for AEs according to type. Indeed, in the case of drugs, the WHO-UMC guidelines are used as an overall assessment tool to assess an AE that occurred after drug administration. Additional guidelines are used to determine causality between the drug treatment and liver injury or Stevens–Johnson syndrome (SJS), both serious AEs of interest [7,8,48]. Liver injury is a known AE related to cytochrome P450, a liver enzyme involved in the metabolism of many drugs [49,50] and the immune response [51], and SJS is a life-threatening, intolerance reaction of the skin [52]. As such, the factors to be confirmed to determine the causality of drugs vary greatly depending on the diseases involved, as well as the symptoms, diagnosis, pathogenesis, and relevance to the drugs administered. Similarly, disease-specific causality assessment tools for AEs that occur after vaccination are necessary. The significance of this study lies in the development of a disease-specific causality assessment tool for GBS, one of the most notable AEFI, by using the basic framework of WHO AEFI guidelines that have already been tested. Our approach to developing a causality assessment guideline for a specific disease is expected to be generalizable to the causality assessment of other AEFI.

### 4.2. Securing the Objectivity of the Clinical Diagnosis of Guillain–Barré Syndrome through Multi-Step Verification

Case ascertainment of GBS is a necessary prerequisite before we proceed causality assessment. However, GBS is a difficult disease to arrive at a differential diagnosis. This is due to the key symptoms of paralysis and muscular weakness that are present in other neurological diseases and the fact that a range of causes have been identified. Therefore, in this study, we developed a checklist for accurate diagnosis by using the diagnostic code from the ICD-10th, prescription details, the BC criteria, and existing damage compensation reports to secure the diagnostic objectivity of GBS. In addition, an advisory panel of 10 neurologists was formed, and surveys were conducted twice to test the validity of the list. As a result, a list that can help to accurately diagnose GBS was developed by combining laboratory data and clinical evaluation. The items included in the list and the process of developing it are expected to be used not only in these guidelines but also for the objective diagnosis of similar neurological diseases in the future.

### 4.3. Recommended Application Method of Guidelines Aligned with Policy

The NIP is implemented as a part of national policy. Consequently, in South Korea, if a person vaccinated through the NIP experiences an AE, the government assesses causality between the AE and the vaccine and determines the level of damage compensation [53,54]. Institutional frameworks and expert committees have already been established in an attempt to maintain the consistency, objectivity, and validity of assessment. However, in cases that required a significant number of professional opinions from diagnosis to determination of the cause, such as GBS, ensuring the consistency of assessment results has been difficult. Thus, our guidelines were developed in association with epidemiologists and the committee, and an application system was proposed to ensure that the guidelines can be used effectively in the actual compensation policy. This indicates that the results of this study can continue to be used, and the guidelines can be immediately implemented in the actual policy. Furthermore, the guidelines are expected to play a role in filling the gaps between stages that may occur in the current compensation system (by the epidemiological survey, the damage investigation team, and the damage compensation committee).

For the guidelines to be properly used, the collection of patient’s medical records, an interview with the physician in charge, and a patient history taken by an epidemiologist are all of paramount importance. This allows confirmation of diagnosis of GBS and gathers additional information to determine whether the AE was triggered by causes other than the vaccination [20]. To this end, the government should organize systematic and specialized curricula and regularly provide epidemiologists with the results of epidemiologic investigations, so that results do not depend on the ability of the individual epidemiologist. Furthermore, academics should prepare standard guidelines to diagnose GBS and actively promote them to neurologists, so that GBS can be diagnosed using the same criteria. If the government, with the support of academia, can establish a system to regularly search for and incorporate new evidence, the use of GBS guidelines may be increased.

This study has some limitations; therefore, cautious interpretation and application are needed. First, the guidelines were developed based on the most recently published evidence at this time. This means unknown factors that cause GBS may remain obscure and undetected. Systematic support will be needed to continuously update these guidelines to include new research in a timely matter. Second, it is difficult to determine if a patient has had a prior infection that may have caused the GBS, increasing the possibility that causality may be incorrectly attributed to the vaccine. Because there is often a time lag between a potential infectious (or vaccine) trigger and the actual occurrence of GBS, this makes it difficult to tell with certainty whether or not an infectious cause could be ruled out. Although we developed these guidelines to collect comprehensive information related to GBS outbreaks, clinical judgments ultimately belong to experts, and only experts can assess the factors to make decisions. To properly use these guidelines, participation of clinical experts is crucial. Therefore, we suggest appropriate education and active promotion of the guidelines.

## 5. Conclusions 

Causality assessment of AEFI is crucial not only to counteract vaccine hesitancy, but also to implement an evidence-based national vaccine policy. Since each AEFI has distinct characteristics, the demand for developing a condition-specific causality assessment guideline is of great need. Our approach to developing the GBS-specific guideline suggests a transparent and scientific process and provides the basic structure applicable to causality assessment of other AEFIs. If the systematic regular updates by clinical experts and the support by the government are sustained, the GBS-specific guideline developed in our study can be used effectively in the actual vaccine policy.

## Figures and Tables

**Table 1:** Case ascertainment of Guillain–Barré syndrome.

**1. Diagnostic code**							
	ICD-10th code						
2. Brighton Collaboration criteria *							
	No.	Brighton Collaboration criteria		Y	N	NA/UJ	
	2.1.	Bilateral AND flaccid weakness of the limbs					
	2.2	Decreased or absent deep tendon reflexes in weak limbs					
	2.3.	Monophasic illness pattern AND interval between onset and nadir of weakness between 12 h and 28 days AND subsequent clinical plateau					
	2.4.	Absence of an identified alternative diagnosis for weakness					
	2.5.	Cytoalbuminologic dissociation (i.e., elevation of CSF protein level above normal values AND CSF total white cell count <50 cells/μL)					
	2.6.	Electrophysiologic findings consistent with GBS					
	All “Yes” for 2.1.-2.4. ⇒ Level 3 All “Yes” for 2.1.-2.4. and for 2.5. or 2.6. ⇒ Level 2 All “Yes” for 2.1.-2.6. ⇒ Level 1 None of the above ⇒ Unable to distinguish						
	Brighton Collaboration diagnostic level result						
**3. Questions to help diagnosis**							
	No.	Examinations to diagnose Guillain–Barré syndrome	Y	N	NA	Note	
	3.1.	Was a test for nerve conduction performed? Write the date of the test and specifics in the note column.					
	3.2.	Was a test for cerebrospinal fluid performed? Write the date of the test and specifics in the note column.					
		Questions to help diagnose Guillain–Barré syndrome	Y	N	NA	UJ	Note
	3.3.	Was the paralysis ascending?					
	3.4.	Did it take less than 4 weeks for symptoms to stop worsening?					
		Questions that might indicate diseases other than Guillain–Barré syndrome					
	3.5.	Were the symptoms accompanied by cognitive deterioration?					
	3.6.	Did the initial symptoms include fever?					
	3.7.	Did the initial symptoms include bowel or bladder dysfunction?					
	3.8.	Was there any other cause of muscular weakness or paralysis?					

* Brighton Collaboration criteria for Guillain–Barré syndrome is quoted and reconstructed from Definition and Application of Terms for Vaccine Pharmacovigilance by Report of CIOMS/WHO Working Group on Vaccine Pharmacovigilance in 2012 on pages 84–85. Y, Yes; N, No; NA, Not applicable; UJ, Unable to judge.

**Table 2:** Causality assessment guidelines for Guillain–Barré syndrome following immunization: Checklist.

**1. Order of the incidence**							
	⚬ Was the vaccine administered before Guillain–Barré syndrome occurred?		Yes/No				
**2. Temporal proximity (time interval)**							
	⚬Did Guillain–Barré syndrome occur within 6 weeks following vaccination?		Yes/No				
	⚬Write the time interval between vaccination and the occurrence of Guillain–Barré syndrome (e.g., 4 weeks following vaccination).						
**3. Evidence for other causes**							
	⚬ For each question, please answer Y(=Yes), N(=No), UK(=Unknown) or NA(=Not applicable). ⚬ Please write the reason for your answer in the note column.						
			**Y**	**N**	**UK**	**NA**	**Note**
	History *	3.1 Has the patient ever experienced Guillain–Barré syndrome or other neuromuscular disorders regardless of vaccination? Please write the name of the disease in the note column.					
		3.2. Has the patient ever been vaccinated with the same vaccine in the past? If so, please write the vaccination date.					
		3.3. Has the patient ever experienced Guillain–Barré syndrome or any other neuromuscular disease after any type of vaccination?					
	Patient’s condition before appearance of GBS *	⚬ Has the patient ever experienced **any items listed below** before the manifestation of Guillain–Barré syndrome? If so, please write the date of the infection.					
		3.4. Upper respiratory infections					
		3.5. GI troubles					
		3.6. Any other symptoms (Zika, Dengue, etc.)					
		3.7. Malaria					
		3.8. Tsutsugamushi					
		3.9. Surgery					
	**Patient examination**	⚬ Was a test for **any pathogens listed below** performed after the manifestation of Guillain–Barré syndrome? If so, write the date of the test, test result, and specific notes.					
		3.10. Campylobacter jejuni					
		3.11. Cytomegalo virus (CMV)					
		3.12. Epstein-Barr virus (EBV)					
		3.13. Herpes Simplex virus (HSV)					
		3.14. Varicella-zoster virus (VZV)					
		3.15. Mycoplasma pneumonia					
		3.16. Hemophilus influenza					
		3.17. Influenza virus					
		3.18. Other pathogens					
	**Immunization anxiety**	3.19. Was the Guillain–Barré syndrome a stress response to vaccination? (e.g., acute stress response, vasovagal syncope, hyperventilation, anxiety, etc.)					
	**Vaccine quality**	3.20. Could the vaccine given to this patient have a quality defect or is substandard or counterfeit?					
	**Immunization errors (please write type of error, if any)**	3.21. Did anything unusual occur during vaccination preparation? (e.g., incorrect mixing, use of expired vaccine, abnormal physical condition, etc.)					
		3.22. Did anything unusual occur during the vaccination procedure? (e.g., Inoculation timing/dose/site/route, needle size error, etc.)					
	⚬ If there are any suspicious causes other than those listed above, write the details below. ⚬ Other causes						
**4. Published evidence (literature, WHO GACVS, IOM etc.) regarding a causal association between the vaccine and GBS**							
	Influenza vaccine						
	Meningococcal vaccine						
	Hepatitis A vaccine						
	Hepatitis B vaccine						
	MMR vaccine						
	HPV vaccine						
	DTP vaccine						
	HiB vaccine						
	Polio vaccine						
	Varicella vaccine						

WHO GACVS, WHO Global Vaccine Safety; IOM, Institute of Medicine. * The time window between prior infection and vaccination was considered as 6 weeks based on a literature review and expert survey.

## Supplementary Materials

The following are available online at https://www.mdpi.com/2076-393X/8/1/101/s1. Supplementary Materials S1: Literature review for potential triggers for the development of Guillain–Barré syndrome. Supplementary Materials S2: Survey questionnaire for the inclusion of potential triggers in causality assessment guidelines of Guillain–Barré syndrome following immunization (English).
