# Supplementary material for: Causality Assessment Guidelines for Adverse Events Following Immunization with a Focus on Guillain–Barré Syndrome
Source: Vaccines (Basel). 2020 Feb 24;8(1):101. doi: 10.3390/vaccines8010101 (PMC7157213; doi:10.3390/vaccines8010101)
Supplement: Supplementary file 1 [file vaccines-08-00101-s001.zip › Supplementary 1.docx]

**Supplementary Material 1.**

**Literature review for potential triggers for the development of Guillain–Barré syndrome**

**Step 1.**

We first searched PubMed for studies written in English using the terms “Guillain–Barré syndrome”[Mesh] or “Guillain–Barré syndrome” on 2019/06/30. The search yielded 8,015 results.

| **Number** | **Search terms (Queries)** | **Results** |
| --- | --- | --- |
| 1 | “Guillain–Barré syndrome”[Mesh] or “Guillain–Barré syndrome”; English | 8015 |

**Step 2.**

We then searched for each potential trigger and combination with the development of Guillain–Barré syndrome (GBS).

|  | **Search terms (Queries)** | **Results** |
| --- | --- | --- |
| **Patient’s condition before appearance of GBS** | | |
| *3.4. Upper respiratory infections* | “Respiratory Tract Infections”[Mesh] OR “upper respiratory infections” AND 1 | 283 |
| *3.5. Gastrointestinal (GI) troubles* | “Gastrointestinal Diseases”[Mesh] OR “Gastrointestinal” OR “GI disease” AND 1 | 335 |
| *3.6. Any other symptoms*  *– Zika/Dengue* | “Zika Virus Infection”[Mesh] OR “Dengue Virus”[Mesh] OR “Arbovirus Infections”[Mesh] OR “Chikungunya virus”[Mesh] AND 1 | 506 |
| *3.7. Malaria* | “Malaria”[Mesh] or “Malaria” AND 1 | 17 |
| *3.8. Tsutsugamushi* | “Scrub Typhus”[Mesh] OR “Orientia tsutsugamushi”[Mesh] OR “scrub typus” OR “Tsutsugamushi” OR “rickettsia” AND 1 | 10 |
| *3.9. Surgery* | “General Surgery”[Mesh] OR “surgery” AND 1 | 1021 |
| **Patient examination for pathogens** | | |
| *3.10. Campylobacter jejuni* | “Campylobacter jejuni”[Mesh] OR Campylobacter jejuni OR “Campylobacter jejuni” OR C. jejuni OR “C. jejuni” OR “Campylobacter” AND 1 | 725 |
| *3.11. Cytomegalo virus (CMV)* | “Cytomegalovirus Infections”[Mesh] OR “Cytomegalovirus”[Mesh] OR “Cytomegalovirus Infections” OR “Cytomegalovirus” OR “CMV” AND 1 | 168 |
| *3.12. Epstein-Barr virus (EBV)* | “Epstein–Barr Virus Infections”[Mesh] OR “Epstein–Barr virus” OR “Epstein–Barr” AND 1 | 115 |
| *3.13. Herpes Simplex virus (HSV)* | “Herpesviridae”[Mesh] OR “herpes simplex” OR “herpes simplex virus” OR herpes simplex AND 1 | 131 |
| *3.14. Varicella-zoster virus (VZV)* | “Varicella Zoster Virus Infection”[Mesh] OR “Varicella Zoster” OR “Varicella-zoster” OR varicella zoster AND 1 | 69 |
| *3.15. Mycoplasma pneumonia* | “Pneumonia, Mycoplasma”[Mesh] OR “mycoplasma pneumonia” OR “mycoplasma” OR mycoplasma pneumonia AND 1 | 93 |
| *3.16. Hemophilus influenza* | “Haemophilus influenza”[Mesh] OR “Haemophilus influenza*” OR “Hemophilus influenza*” OR hemophilus influenza OR haemophilus influenza AND 1 | 37 |
| *3.17. Influenza virus* | “Influenza, Human”[Mesh] OR “influenza virus” OR “influenza infection” OR influenza AND 1 | 350 |

**Step 3.**

A two-step eligibility assessment was performed independently by two researchers (S. Cho and A. Kim) for all the search results of each subject, by screening the titles and abstracts. Studies without full-text articles or no information about evidence on biological mechanisms or the time periods to develop GBS were excluded.

**Step 4.**

The final studies included were reviewed by neurologists. If the results of studies overlapped, we selected a review or the latest study and then qualitatively synthesized them as below.

|  | **Evidence on biological mechanisms and time periods** | **Source** |
| --- | --- | --- |
| **Patient’s condition before the appearance of GBS** | | |
| *3.4. Upper respiratory infections* | - Infectious symptoms occurred 3 weeks or less before neuropathic onset in most of the GBS patients, whereas the latent period was often longer than 3 weeks in the control groups. | Koga, 2001 |
| *3.5. Gastrointestinal (GI) troubles* | - Because evidence for a recent C. jejuni infection was found in 8 of 10 recently collected serum samples, this pathogen likely is the predominant cause of gastroenteritis. | Van Koningsveld, 2001 |
| *3.6. Any other symptoms*  *– Zika/Dengue* | - It is possible that the development of GBS is related to molecular mimicry, where virus specific antibodies (ZIKV or DENV) cross-react with nerve-cells, but it is also possible that this association between neutralizing antibodies and GBS results from indirect effects such as a high virus load or high immune activation. | Lynch, 2019 |
| *3.7. Malaria* | - In malaria, the asexual stage of infection is accompanied by the release of cytokines and other immunological mediators. These immunological mediators may trigger inflammation of the axons, causing demyelination. - The malaria parasite may damage peripheral nerves by vascular occlusion, causing anoxemic stagnation in the vasa nervosum, leading to temporary demyelination and recovery after disappearance of parasitemia and the establishment of normal blood flow in the vasa nervosum. | Gangula, 2017  Wijesundere, 1992 |
| *3.8. Tsutsugamushi* | - Mimicry involves the sharing of antigens between the host and infecting microorganism, and it is a type of cross-reactivity similar to that of an autoimmune disease. It is enabled by the activation of receptors on B- or T-lymphocytes by the host and microorganism. | Ju, 2011 |
| *3.9. Surgery* | - The etiology of peripheral neuropathy after bariatric surgery is probably multifactorial, with nutritional deficiencies being the most common etiology. The pathophysiology of GBS has otherwise been reported to be immunogenic. - The time interval between surgery and GBS onset also has been studied. Duncan and Kennedy stated that postsurgical GBS generally occurs 2-3 weeks after the operation. | Ishaque, 2015  Qi, 2018 |
| **Patient examination for pathogens** | | |
| *3.10. Campylobacter jejuni* | - C. jejuni ignites autoimmunity as a result of molecular mimicry between C. jejuni lipooligosaccharides (LOS) and host gangliosides. There is also an active role for cell-mediated and innate immunity in the pathogenesis of GBS. - C. jejuni strains isolated from GBS patients have a LOS with a GM1-like structure. Molecular mimicry between LOS and the peripheral nerves as a cause of GBS was demonstrated in animal models of human GBS. | Ebrahim, 2019  Islaeil, 2012 |
| *3.11. Cytomegalo virus (CMV)* | - Molecular mimicry of LOSs on the surface of infectious agents and ganglioside antigens on neural cells is thought to induce cross-reactive humoral and cellular immune responses. Patients with GBS develop antibodies against those gangliosides, resulting in autoimmune targeting of peripheral nerve sites, leading to neural damage. - Based on an electrodiagnostic study, patients with cytomegalovirus or Epstein–Barr virus-related AIDP (acute inflammatory demyelinating polyneuropathy) showed progressive increases in distal latencies in the 8 weeks after onset. | Kaida, 2009  Kuwabara, 2004 |
| *3.12. Epstein–Barr virus (EBV)* | - EBV can directly infiltrate the peripheral nervous system, usually resulting in focal or multifocal neuritis rather than polyneuropathy. Neuropathological studies have revealed perivascular lymphocytic infiltrates, parenchymal edema, microglial proliferation, and inflammatory demyelinating lesions. Further, in situ hybridization using EBV-encoded small RNA-1 (EBER1) did not yield positive signals, suggesting that demyelination with secondary axonal degeneration occurs without infiltration of EBV-infected lymphocytes. - Based on an electrodiagnostic study, patients with cytomegalovirus or EBV-related AIDP (acute inflammatory demyelinating polyneuropathy) showed progressive increases in distal latencies in the 8 weeks after onset. | Rodriguez, 2018  Kuwabara, 2004 |
| *3.13. Herpes Simplex virus (HSV)* | - Inflammatory nerve injury caused by cross-reactive antibodies to HSV-1 and 2 through molecular mimicry is the most likely mechanism of GBS. - About 75% of patients with GBS have a history of preceding infection, usually of the respiratory and gastrointestinal tract, in the 6 weeks prior to onset. | Rodriguez, 2018 |
| *3.14. Varicella-zoster virus (VZV)* | - Current evidence suggests VZV actively remodels T cells into activated specific tissue-homing infected T cells. However, whether this leads to autoimmunity against nerves has yet to be determined. In addition, VZV can directly infect the peripheral nerves, which may produce the neurophysiologic features of peripheral nerve demyelination. However, polyradiculoneuritis due to infection of nerve roots and/or nerves by VZV is unlikely, since weakness did not coincide with the skin rash in any case and none of the patients had an increased CSF cell count. Immune-mediated demyelination is also possible in other acute and mono or polyphasic neurological disorders preceded by VZV infection. VZV may also potentially trigger giant cell arteritis (GCA), a form of autoimmune vasculopathy with the pathophysiology further indicating VZV-mediated macrophase and T cell-mediated inflammation. - In the case of GBS following chicken pox or varicella, the duration between the skin rash and developing weakness ranged from 4 to 21 days. In the case of GBS following herpes zoster or shingles, the duration between the skin rash and developing weakness was 3–42 days. | Islam, 2018 |
| *3.15. Mycoplasma pneumonia* | - These results suggest that the development of anti‐GalC IgG is a critical step in the pathogenesis of GBS after an infection with M. pneumoniae. - The immunologic pathomechanism of GBS in association with the M. pneumoniae infection has been suggested to involve anti-galactocerebroside and anti-ganglioside antibodies. Susuki et al. reported that M. pneumoniae carries both the ganglioside GM1 and galactocerebroside epitopes, and the antibody to GM1 might be more pathogenic than that to galactocerebroside. | Meyer, 2016  Narita, 2009- |
| *3.16. Hemophilus influenza* | - Having both IgG1 and IgG3 antibodies was related to upper respiratory tract infections and cross-reactive antibodies to Hemophilus influenzae lipo-oligosaccharides. - Of 11 patients with high titers of the IgG antibody against H. influenza type B, a respiratory tract infection 4 weeks before GBS onset occurred in 8 (72.7%) and gastrointestinal infection occurred in 2 (18.2%). | Jacobs, 2008  Nafissi, 2013 |
| *3.17. Influenza virus* | - Anti-GQ1b antibodies are the most frequently detected antibodies in GBSRD-I GBS-related diseases after influenza virus infection). - A study from Germany on the Pandemrix vaccine reported a statistically significant association between GBS and vaccination in a 5–42 day risk window. | Yamana, 2019  Ghaderi, 2016 |

**References**

Ebrahim, Soltani Z.; Rahmani, F.; Rezaei, N. Autoimmunity and cytokines in Guillain-Barré syndrome revisited: review of pathomechanisms with an eye on therapeutic options. European cytokine network. 2019, 30(1), 1-14.

Gangula, Rahul Sai, et al. "Guillain-Barre syndrome with falciparum malaria and scrub typhus mixed infection-an unusual combination." Journal of clinical and diagnostic research. 2017, 11(9), OD10.

Ghaderi, S; Gunnes, N; Bakken, IJ, Magnus, P; Trogstad, L; Håberg, SE. Risk of Guillain-Barré syndrome after exposure to pandemic influenza A(H1N1)pdm09 vaccination or infection: a Norwegian population-based cohort study. European journal of epidemiology. 2016, 31(1),67-72.

Ishaque, Noman, et al. "Guillain–Barré syndrome (demyelinating) six weeks after bariatric surgery: a case report and literature review." Obesity research & clinical practice. 2015, 9(4), 416-419.

Islam, B; Islam, Z; GeurtsvanKessel, CH; Jahan, I; Endtz, HP; Mohammad, QD; Jacobs, BC. Guillain-Barré syndrome following varicella-zoster virus infection. European journal of clinical microbiology & infectious diseases. 2018, 37(3), 511-518.

Israeli, E; Agmon-Levin, N; Blank, M; Chapman, J; Shoenfeld, Y. Guillain-Barré syndrome--a classical autoimmune disease triggered by infection or vaccination. Clinical reviews in allergy & immunology. 2012, 42(2), 121-30.

Jacobs, BC; Koga, M; van Rijs, W; Geleijns, K; van Doorn, PA; Willison, HJ; Yuki N. Subclass IgG to motor gangliosides related to infection and clinical course in Guillain-Barré syndrome. Journal of neuroimmunology. 2008, 194(1-2),181-90.

Ju, Il Nam, et al. "Two cases of scrub typhus presenting with Guillain-Barré syndrome with respiratory failure." The Korean journal of internal medicine. 2011, 26(4), 474.

Kaida, K; Ariga, T; Yu, RK. Antiganglioside antibodies and their pathophysiological effects on Guillain-Barré syndrome and related disorders--a review. Glycobiology. 2009, 19(7), 676-92.

Koga, M., N. Yuki, and K. Hirata. "Antecedent symptoms in Guillain–Barré syndrome: an important indicator for clinical and serological subgroups." Acta Neurologica Scandinavica. 2001, 103(5), 278-287.

Kuwabara, S. Guillain-Barré syndrome: epidemiology, pathophysiology and management. Drugs. 2004, 64(6), 597-610.

Lynch, Rebecca M., et al. "Augmented Zika and dengue neutralizing antibodies are associated with Guillain-Barre syndrome." The Journal of infectious diseases. 2019, 219(1), 26-30.

Meyer Sauteur PM; Huizinga R; Tio-Gillen AP; Roodbol J; Hoogenboezem T; Jacobs E; van Rijn M; van der Eijk AA; Vink C; de Wit MC; van Rossum AM; Jacobs BC. Mycoplasma pneumoniae triggering the Guillain-Barré syndrome: A case-control study. Annals of neurology. 2016, 80(4), 566-80.

Nafissi, S; Vahabi, Z; Sadeghi Ghahar, M; Amirzargar, AA; Naderi, S. The role of cytomegalovirus, Haemophilus influenzae and Epstein Barr virus in Guillain Barre syndrome. Acta medica iranica. 2013, 51(6),372-6.

Narita, M. Pathogenesis of neurologic manifestations of Mycoplasma pneumoniae infection. Pediatric neurology. 2009, 41(3), 159-66.

Qi, Bao, and Chunyang Meng. "Retrospective Analysis and Comment on the Time Interval Between Surgery and Onset of Guillain-Barré Syndrome." World neurosurgery. 2018, 109, 499.

Van Koningsveld, R., et al. "Gastroenteritis-associated Guillain–Barre syndrome on the Caribbean island Curacao." Neurology. 2001, 56(11), 1467-1472.

Wijesundere, Anula. "Guillain-Barré syndrome in Plasmodium falciparum malaria." Postgraduate medical journal. 1992, 68.799, 376.

Y, Rodriguez; M, Rojas; Y, Pacheco; Y, Acosta-Ampudia; C, Ramirez-Santana; D.M. Monsalve; M.E. Gershwin; J.-M. Anaya, Guillain-Barre syndrome, transverse myelitis and infectious diseases. Cellular and molecular immunology. 2018, 15, 547-562.

Yamana. M; Kuwahara. M; Fukumoto, Y; Yoshikawa, K; Takada, K; Kusunoki, S. Guillain-Barré syndrome and related diseases after influenza virus infection. Neurology(R) neuroimmunology & neuroinflammation. 2019, 6(4), e575.
