# Supplementary material for: Causality Assessment Guidelines for Adverse Events Following Immunization with a Focus on Guillain–Barré Syndrome
Source: Vaccines (Basel). 2020 Feb 24;8(1):101. doi: 10.3390/vaccines8010101 (PMC7157213; doi:10.3390/vaccines8010101)
Supplement: Supplementary file 1 [file vaccines-08-00101-s001.zip › Supplementary 2.docx]

**Supplementary Material 2.**

**2-1. Survey questionnaire for the inclusion of potential triggers in causality assessment guidelines of Guillain–Barré syndrome following immunization (English)**

Potential triggers (possible causes) of Guillain–Barré syndrome have been investigated as listed below. Do you think each trigger is appropriate to be included in the causality assessment guideline following immunization as a cause for Guillain–Barré syndrome? If you think it is appropriate, please mark √ in “appropriate” or if it’s not appropriate, please mark √ in “inappropriate” and describe the reason for your choice. Please see the attached evidence-based sources related to each item (**Supplementary Material 1**).

| Potential triggers for the development of GBS | | Is it appropriate to be included in the causality assessment guidelines for GBS? | | | **<Time period>**  When do you think each trigger (possible cause), which can be suspected in GBS development, must appear before GBS is diagnosed?  *(E.g., upper respiratory infection before the symptoms of GBS; Upper respiratory tract infection can be presumed to be the cause of GBS if it was within 1 week.)* |
| --- | --- | --- | --- | --- | --- |
|  |  | Appropriate | Inappropriate | Reason for choice |  |
| **Patient’s condition before appearance of GBS** | | | | | |
| 1 | *Upper respiratory infections* |  |  |  |  |
| 2 | *Gastrointestinal (GI) troubles* |  |  |  |  |
| 3 | *Zika/Dengue* |  |  |  |  |
| 4 | *Malaria* |  |  |  |  |
| 5 | *Tsutsugamushi* |  |  |  |  |
| 6 | *Surgery* |  |  |  |  |
| **Examination for pathogens** | | | | | |
| 7 | *Campylobacter jejuni* |  |  |  |  |
| 8 | *Cytomegalo virus (CMV)* |  |  |  |  |
| 9 | *Epstein–Barr virus (EBV)* |  |  |  |  |
| 10 | *Herpes Simplex virus (HSV)* |  |  |  |  |
| 11 | *Varicella-zoster virus (VZV)* |  |  |  |  |
| 12 | *Mycoplasma pneumonia* |  |  |  |  |
| 13 | *Hemophilus influenza* |  |  |  |  |
| 14 | *Influenza virus* |  |  |  |  |

| - If you have other possible causes to add, please write them below. |
| --- |

**2-2. Results of Survey by an expert panel comprising 10 neurologists to assess the quality of evidence about potential triggers (possible causes) (English)**

| Potential triggers for the development of GBS | | Is it appropriate to be included in the causality assessment guidelines for GBS? | | | **<Time period>**  When do you think each trigger (possible cause), which can be suspected in GBS development, must appear before GBS is diagnosed? (unit: weeks) | | | | | | | | | | |
| --- | --- | --- | --- | --- | --- | --- | --- | --- | --- | --- | --- | --- | --- | --- | --- |
|  |  | Appropriate | Inappropriate | **Decision** | A | B | C | D | E | F | G | H | I | J | **Decision^*^** |
| **Patient’s condition before appearance of GBS** | | | | | | | | | | | | | | | |
| 1 | *Upper respiratory infections* | 10 | 0 | Included | 2 | 6 | 4 | 6-8 | 4 | 2 | 3 | 6 | 2-3 | 2-4 | **6** |
| 2 | *Gastrointestinal (GI) troubles* | 10 | 0 | Included | 2 | 6 | 4 | 6-8 | 4 | 2 | 3 | 8 | 2-3 | 2-3 | **6** |
| 3 | *Zika/Dengue* | 10 | 0 | Included | 1 | 6 | 2 | 6-8 | 4 | 2 | 3 | 4 | 2-3 | 4 | **6** |
| 4 | *Malaria* | 9 | 1 | Included | 3 | 6 | UR | UR | 4 | 2 | UR | 8 | 2-3 | 2 | **6** |
| 5 | *Tsutsugamushi* | 9 | 1 | Included | 2 | 6 | 2 | 6-8 | 4 | 2 | UR | 4 | 2-3 | 2 | **6** |
| 6 | *Surgery* | 7 | 3 | Included | 2 | 6 | 8 | UR | 6 | 4 | UR | 6 | UR | 2 | **6** |
| **Patient’s condition before appearance of GBS** | | | | | | | | | | | | | | | |
| 7 | *Campylobacter jejuni* | 10 | 0 | Included | 2 | 6 | 8 | 6-8 | 4 | 2 | UR | 4 | 6 | UR | **6** |
| 8 | *Cytomegalo virus (CMV)* | 10 | 0 | Included | 2 | 6 | UR | 6-8 | 4 | UR | UR | UR | 6 | UR | **6** |
| 9 | *Epstein–Barr virus (EBV)* | 10 | 0 | Included | 2 | 6 | 4 | 6-8 | 4 | UR | UR | 4 | 6 | UR | **6** |
| 10 | *Herpes Simplex virus (HSV)* | 9 | 1 | Included | 2 | 6 | 8 | 6-8 | 4 | UR | UR | 6 | UR | UR | **6** |
| 11 | *Varicella-zoster virus (VZV)* | 10 | 0 | Included | 2 | 6 | 4 | 6-8 | 4 | UR | UR | 4 | 6 | UR | **6** |
| 12 | *Mycoplasma pneumonia* | 10 | 0 | Included | 2 | 6 | 2 | 6-8 | 4 | 2 | UR | 4 | 6 | UR | **6** |
| 13 | *Hemophilus influenza* | 10 | 0 | Included | 2 | 6 | UR | 6-8 | 4 | 2 | UR | 4 | 6 | UR | **6** |
| 14 | *Influenza virus* | 10 | 0 | Included | 2 | 6 | 8 | 6-8 | 4 | 2 | UR | 4 | 6 | UR | **6** |

UR, Unable to respond

**^*^**The final decision was made by the panel of Epidemiology Intelligence Service officers and members of Vaccine Injury Compensation Expert Committee.
